# Verbal memory functioning in recurrent depression during partial remission and remission-Brief report

**DOI:** 10.3389/fpsyg.2013.00652

**Published:** 2013-10-08

**Authors:** Åsa Hammar, Guro Årdal

**Affiliations:** ^1^Department of Biological and Medical Psychology, University of BergenBergen, Norway; ^2^Division of Psychiatry, Haukeland University Hospital, University of BergenBergen, Norway; ^3^Moodnet Research Group, Haukeland University Hospital, University of BergenBergen, Norway

**Keywords:** verbal memory, depression, cognitive function, partial remission, remission

## Abstract

The aim of the present study was to investigate verbal memory performance in a group of patients with remitted and partial remitted major depressive disorder. Thirty-one patients and 31 healthy matched controls were included in the study. Results from the California Verbal Learning Test show intact verbal memory performance in the patient group regarding learning, recall and recognition. However, patients had significantly poorer performance compared to healthy controls in immediate recall of the first trial in the verbal memory test. In conclusion, the patient group showed intact memory performance, when material is presented more than once. These findings indicate that memory performance in MDD patients with partial remission and remission benefit from repetition of material.

## Introduction

Depressed patients often report generalized memory problems, however it has been shown that they estimate their actual performance worse than objective measures (Kalska et al., 1999; Wong et al., 2000). Indeed, several studies show intact memory functioning in depression (Egeland et al., 2003; Porter et al., 2003; Smith et al., 2005; Wang et al., 2006; Hammar et al., 2011). In contrast, others report memory impairments during acute phase of the disorder (for overview se Austin et al., 2001; Fossati et al., 2002, 2004; Neu et al., 2005; Bearden et al., 2006). Hence, the literature regarding acute depression and memory impairment is divergent and inconclusive. Consequently, the long term course of verbal memory functioning during phases of symptom recovery is still unclear.

Nevertheless, in the comprehensive review conducted by Douglas and Porter (2009) it was concluded that whilst some cognitive functions remain impaired during symptom improvement it seems as functioning in verbal memory recovers. Still, the number of published reports that investigates verbal memory in a long term course are few.

In a recent study by Hammar et al. (2011), a group of patients diagnosed with unipolar recurrent major depression was investigated in verbal memory. All patients were in an acute phase of depression with severe symptom load. We found intact verbal learning and memory, however the patient group reported significantly fewer words compared to the healthy control group on the first immediate recall trial in the verbal learning test. Performance of the patients were normalized during the remaining trials. In order to pursue the finding regarding first trial impairment, we conducted a study including patients previously diagnosed with recurrent major depressive disorder and currently in remission or partial remission.

Based on the previous literature we hypothesized that patients with recurrent MDD would show normal performance on the verbal memory test and that the impaired performance regarding the first trial would be normalized.

## Methods

### Subjects

Data from 31 patients (11 males, 20 females) who formerly met the DSM-IV criteria (DSM-IV, 2000) for a unipolar recurrent MDD diagnosis, using MINI - International Psychiatric Structural Interview (Leiknes et al., 1999) and 31 healthy matched controls were included in the present study. Severity of depression was assessed using the Hamilton Depression Rating Scale (HDRS), (Hamilton, 1960). For demographic data see Table 2.

The patient group had experienced a minimum of two previous episodes of MDD, with at least one episode scoring a minimum of 18 on HDRS and at testing the patients had experienced improvement in symptoms of depression. Patients have been excluded from the study if known; history of brain damage, alcohol and/or substance abuse.

The control group was matched to the patient group ± a 2 year limit on age, and completed years of education. Exclusion criteria on control subjects were alcohol or any other substance abuse, brain damage, or a history of any mental disorder.

### Procedure

All participants were assessed regarding their learning and memory capacity using the California Verbal Learning Test (CVLT) (Delis et al., 1987). CVLT is a verbal learning and memory test, which permits examination of acquisition, retrieval, and retention processes (Basso and Bornstein, 1999).The CVLT-II measures both recall and recognition over five immediate trials and five delayed memory trials, using two word lists consisting of 16 words each. See Table 1 for an overview of obtained variables.

**Table 1:** **CVLT-II variables**.

**Trial**	**Name**	**Description**
1	Immediate free recall	Number of words recalled from list A in the first trial.
1–5	Level of learning	Total number of words recalled in the first five trials.
7	Short delay free recall	Number of words recalled from list A after list B was presented.
8	Short delay cued recall	Number of words recalled from list A with categories as cues.
9	Long delay free recall	Number of words recalled from list A after 20 min delay.
10	Long delay cued recall	Number of words recalled from list A with categories as cues.
11	Recognition total hits	Number of words recognized from list A (yes/no).

Informed consent was obtained from all participants. The study was performed in accordance with the Helsinki Declaration of the World Medical Association Assembly. The study was approved by the Regional Committee for Medical Research Ethics and The Norwegian Data Inspectorate.

### Data scoring and analysis

The statistical analysis of the data was carried out using the statistical package SPSS (20.0 for Windows). Group comparison analyses were carried out using a one way ANOVA. Group comparison analyses were carried out using a one way ANOVA. An alpha level of < 0.05 was used for statistical tests. Age and level of education were controlled for through the matched control group. Preliminary assumption testing was conducted to check for normality, linearity and outliers. In order to check for equal variance, Levine's test of homogeneity of variance was conducted. To measure effect sizes for significant differences partial eta square was calculated. Data were analyzed as number of words recalled in the different conditions or as number of words correctly recognized. An independent samples *t*-test was conducted to compare the IQ scores for the patient and control group. Correlations were calculated with Pearson's coefficients.

## Results

CVLT: Immediate recall (trial 1): The patient group recalled significant fewer words compared to the control group on immediate recall, trial 1 *F*_(1, 60)_ = 6.183, *p* = 0.016. Partial eta squared = 0.09. There were no significant differences between the two groups in the other conditions (see Table 2).

**Table 2:** **Test performance and descriptive data for both groups**.

	**Patient group**		**Control group**		***df***	***F***	**Sig.**
	***M***	***SD***	***M***	***SD***			
**CVLT**							
Immediate recall (trial 1)	7.5	1.8	8.7	2.2	1.60	6.2	^*^
Level of learning (trial 1–5)	55.3	9.3	59.2	11.1	1.60	2.3	*p* = 0.13
Short delay free recall	12.4	2.8	12.6	3.0	1.60	0.09	*p* = 0.76
Long delay free recall	12.9	2.7	13.3	3.0	1.60	0.24	*p* = 0.63
Recognition total hits	15.4	0.9	15.5	0.8	1.60	0.21	*p* = 0.65
**DESCRIPTIVE DATA**							
Age	43.5	12.5	45.8	11.6	1.60	0.31	*p* = 0.46
Education	14.1	3.1	13.6	3.2	1.60	0.27	*p* = 0.52
IQ (WASI)	107.5	14.6	115.2	9.9	1.60	3.3	^*^
HDRS	8.03	5.0	N.A	N.A			

* Significant at p > 0.05.

**Figure 1:**
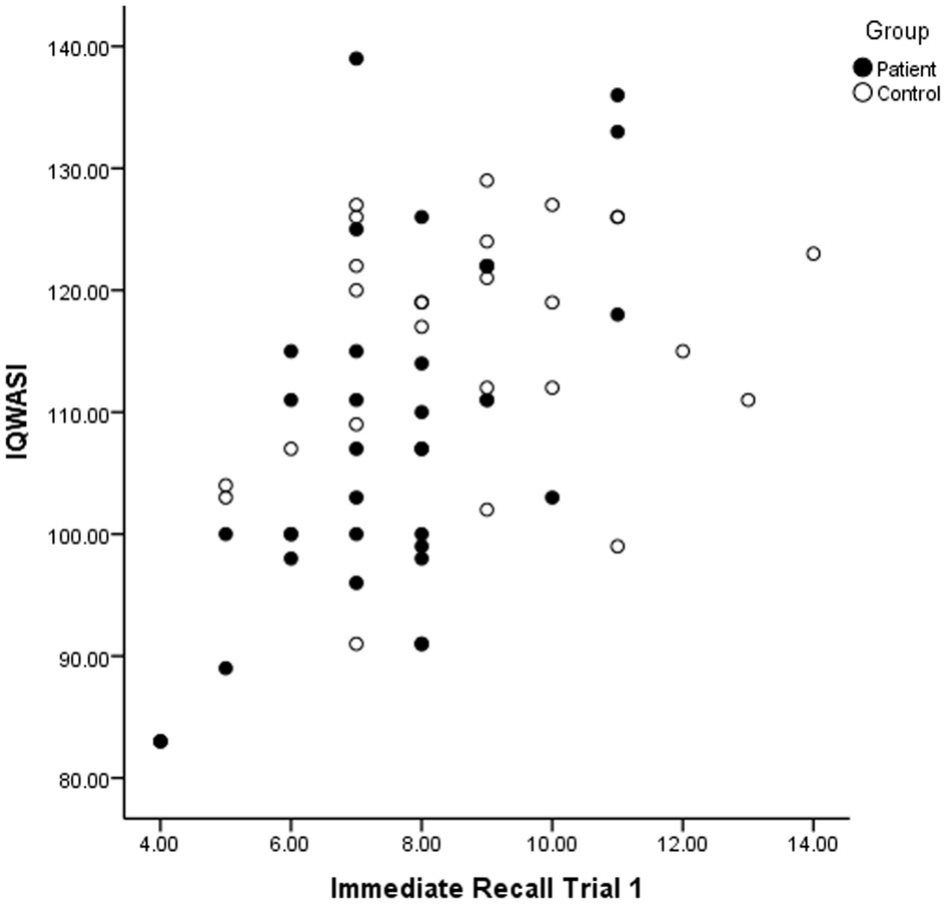
**The correlation between IQ and Immediate Recall for patients and controls**.

There was no correlation between current symptoms of depression (as measured by HDRS) and performance on immediate recall, trial 1. There was a significant difference between the patient and the control group on WASI total IQ score *t*_(60)_ = −2,43, *p* = 0.018. Further there was a significant correlation in the patient group *r* = 0.575, *n* = 31, *p* > 0.01 between WASI total IQ score and scores on immediate recall, trial 1. There was no correlation between WASI total IQ score and immediate recall, trial 1 in the control group. See Figure 1.

To further explore the possible link between current depressive symptoms and performance on immediate recall, trial 1 the patient group was divided into two groups: remitted (*N* = 13) and partial remitted (*N* = 18) based on HDRS scores. Remission was defined as a HDRS score of ≥ 7 and partial remission was defined as ≤ 8 and improvement from baseline inclusion.

There were no significant difference in immediate recall, trial 1 between patients in remission and patients with partial remission (see Table 3).

**Table 3:** **Test performance and descriptive data for the remission group (*N* = 18) and the partial remission group (P.Rem. group) (*N* = 13)**.

	**Remission group**		**P.Rem. group**		***df***	***F***	Sig. ^*^
	***M***	***SD***	***M***	***SD***			
**CVLT**							
Immediate recall (trial 1)	7.4	1.9	7.6	1.7	1.29	0.18	*p* = 0.67
Level of learning (trial 1–5)	53.5	10.5	57.7	7.0	1.29	1.6	*p* = 0.22
Short delay free recall	12.0	3.1	12.8	2.4	1.29	0.66	*p* = 0.42
Long delay free recall	12.3	3.2	13.7	1.5	1.29	2.0	*p* = 0.16
Recognition total hits	15.3	1.1	15.5	0.05	1.29	0.65	*p* = 0.42

* Significant at p > 0.05.

## Discussion

Patients with remitted and partial remitted major depression show intact verbal memory performance regarding learning, recall and recognition in the present study. The patient group had significantly poorer performance compared to the control group in immediate recall of the first trial in verbal memory.

Thus, the hypothesis that patients with recurrent MDD would show normal performance on the verbal memory test was confirmed, however, the expectation of normalized performance on immediate recall of the first trial was not supported. Severity of depression did not have any effects of the results.

There are various possible explanations for the findings in the present study. As described in the CVLT manual (Delis et al., 1987) depressed patients might struggle on the first learning trial because they feel overwhelmed, thus performance will normalize trough the test. Such an explanation is in accordance with previous findings in acutely depressed patients (Hammar et al., 2011). According to Delis et al. (2000) the first immediate recall trial on the CVLT-II is thought to be especially dependent on auditory attention span. Individuals with impaired attention, but normal learning and memory may perform poorly on the first immediate recall trial, but perform adequately on subsequent trials (Delis et al., 2000). This is a plausible explanation for the findings in the present study, since attention is a domain which often is impaired in depressed patients (Hammar and Årdal, 2009). Deficits in attention have been reported in studies of remitted patients (Weiland-Fiedler et al., 2004; Paelecke-Habermann et al., 2005; Preiss et al., 2009).

The patient group was divided into patients in remission or partially remission in order to investigate if performance was related to symptom load. There was no difference in performance on immediate recall of the first trial between the two groups. Moreover, there was no correlation between symptom load and performance on the first learning trial. Thus, the results in the present report could not be explained by the presence of depressive symptoms.

In a clinical perspective the finding reflects the often self-reported experience of poor memory functioning in this patient group (Kalska et al., 1999; Wong et al., 2000), since daily life memory functioning often requires immediate recall without repetition. When disseminating these results, it is of importance to communicate both the cognitive strengths in verbal memory in this patient group, but at the same time not underestimate the dysfunction found in immediate recall of the first trial.

A correlation between IQ scores and immediate recall was observed in immediate recall of the first trial in the patient group, indicating that higher IQ is associated with better performance on immediate recall. There was no correlation between these measures in the control group. This finding was not evident in a previous study of patients in the acute phase of depression (Hammar et al., 2011). High IQ seems to benefit improved performance in immediate recall, when patients are in remission or partial remission. However this seems not to be the case in the acute phase of illness (Hammar et al., 2011). It is important to note that IQ was average in the patient group, thus low IQ could not explain the result in general.

Little research is published showing cognitive strengths in depressed patients. Following the reasoning by Delis et al. (2000), the present finding of impairment in immediate recall in the patient group might reflect impaired attention span rather than memory impairment. However, it is important to highlight the intact learning performance when repetitions are provided, and further the intact short and long-term memory. This knowledge is important for clinicians and the patients themselves in order to plan rehabilitation and interventions. Future research should aim to clarify the impact of attention span on memory functioning with subjective and objective measures in this patient group.

### Conflict of interest statement

The authors declare that the research was conducted in the absence of any commercial or financial relationships that could be construed as a potential conflict of interest.
